# Prenatal Exposure to an EDC Mixture, NeuroMix: Effects on Brain, Behavior, and Stress Responsiveness in Rats

**DOI:** 10.3390/toxics10030122

**Published:** 2022-03-03

**Authors:** Andrea C. Gore, Tatum Moore, Matthew J. Groom, Lindsay M. Thompson

**Affiliations:** Division of Pharmacology & Toxicology, College of Pharmacy, The University of Texas at Austin, Austin, TX 78712, USA; tatummoore@utexas.edu (T.M.); matt.groom@bath.edu (M.J.G.); lindsay.thompson82@utexas.edu (L.M.T.)

**Keywords:** endocrine-disrupting chemical (EDC), behavior, stress, development, mixture, NeuroMix, BPA, BPS, PCB, phthalate, PBDE, PFOS

## Abstract

Humans and wildlife are exposed to endocrine-disrupting chemicals (EDCs) throughout their lives. Environmental EDCs are implicated in a range of diseases/disorders with developmental origins, including neurodevelopment and behavior. EDCs are most often studied one by one; here, we assessed outcomes induced by a mixture designed to represent the real-world situation of multiple simultaneous exposures. The choice of EDCs, which we refer to as “NeuroMix,” was informed by evidence for neurobiological effects in single-compound studies and included bisphenols, phthalates, vinclozolin, and perfluorinated, polybrominated, and polychlorinated compounds. Pregnant Sprague Dawley rats were fed the NeuroMix or vehicle, and then offspring of both sexes were assessed for effects on postnatal development and behaviors and gene expression in the brain in adulthood. In order to determine whether early-life EDCs predisposed to subsequent vulnerability to postnatal life challenges, a subset of rats were also given a stress challenge in adolescence. Prenatal NeuroMix exposure decreased body weight and delayed puberty in males but not females. In adulthood, NeuroMix caused changes in anxiety-like, social, and mate preference behaviors only in females. Effects of stress were predominantly observed in males. Several interactions of NeuroMix and stress were found, especially for the mate preference behavior and gene expression in the brain. These findings provide novel insights into how two realistic environmental challenges lead to developmental and neurobehavioral deficits, both alone and in combination, in a sex-specific manner.

## 1. Introduction

An increasing number of endocrine-disrupting chemicals (EDCs) are associated with neurodevelopmental disorders. Animal models of the “developmental origins of health and disease” (DOHaD) hypothesis show cause-and-effect relationships between early life exposures to individual EDCs and abnormalities in cognitive, affective, and social behaviors later in life [1,2,3,4,5,6,7,8,9]. Epidemiological evidence in humans suggests that those with higher body burdens of EDCs may have a greater propensity for aberrations in these same behaviors [10,11,12,13,14]. Specific to anxiety-like disorders, these relationships have been shown, to date, for bisphenol A (BPA) [15,16,17,18], phthalates such as DEHP [19,20], and persistent organic pollutants such as polychlorinated biphenyls (PCBs) [21,22] in both rodent and human studies.

Beyond this “one chemical at a time” approach is growing evidence that mixtures of EDCs may have effects that cannot be predicted from outcomes based on single chemical testing. This means that doses of EDCs below the estimated no observed adverse effect level (NOAEL) for an endpoint may have adverse effects when given in combination [23,24,25,26,27,28,29]. This is a closer representation of real-world exposure compared to previous models and highly relevant to humans who are ubiquitously exposed to many chemicals [30,31,32].

EDCs are not the only environmental insult, and their effects may be influenced by other life stressors, including stress. Similar to EDCs, stress experiences during critical periods of development in neonates and adolescents can predispose them to neurobehavioral disorders later in life [33,34]. Stressors can also interact across different developmental stages, a concept illustrated by “two hit” models of psychosocial stress or EDC exposure, in which an initial hit, usually perinatal, is followed by a second hit in adolescence. For example, our group reported that two hits of PCBs, the first gestational and the second in juvenile life, changed gene expression profiles and behaviors in a sexually dimorphic manner [21,35]. Rats that were given neonatal maternal separation followed by a pharmacological stressor (injection of a glucocorticoid, corticosterone) had changes in expression of genes and in behaviors (anhedonia and cognitive) in a manner not predicted by single hits [36,37]. To our knowledge, the only study similar to the current one in which the first hit is an EDC exposure, followed by the second hit of behavioral stress, was published by Panagiotidou et al. The authors showed that BPA exposure through gestational/lactational exposure interacted with stress (forced swimming) in late adolescence in rats on measures of hypothalamic-pituitary-adrenal activity was affected differently by the two hits compared to the individual hits [38]. Beyond that latter study, knowledge about how EDCs and other life stressors interact is largely deficient.

In this study, we tested the hypothesis that NeuroMix would result in physiological and behavioral changes and that stress in adolescence would alter the manifestation of these effects. Therefore, we assessed the effects of prenatal exposure to a low dose EDC mixture, which we refer to as NeuroMix, comprising chemicals selected for known neurobiological effects, on postnatal development and adult behavior. In addition, the interactions of the prenatal EDCs with a chronic stressor through adolescence were also evaluated to ascertain how different common environmental insults influence one another.

## 2. Materials and Methods

### 2.1. Animals and Husbandry

All animal protocols were conducted in accordance with the Guide for the Care and Use of Laboratory Animals and approved by the University of Texas at Austin Institutional Animal Care and Use Committee. Adult (~90 day) female and male Sprague Dawley rats were purchased from Envigo, Indianapolis, IN to serve as breeders for experimental rats. They were housed in standard-sized polysulfone cages in a room that was temperature (21 C) and humidity (51%) controlled. The light–dark cycle was 10 h/14 h, with the lights off at 1100 am. The cages contained Sani-Chip bedding. Rats were acclimated to the light cycle and facility for 2 weeks prior to breeding, during which they were fed a low phytoestrogen (Envigo Teklad) diet. Mating was conducted on the day of anticipated receptivity (determined by vaginal smears) and observed to confirm mating. Then, the pair were left together in the cage overnight, and the female was checked for the presence of sperm to confirm successful mating. The day of mating was termed embryonic day 0 (E0). During gestation from E8 through E18, dams were weighed and fed either the NeuroMix (*n* = 14 dams) or vehicle (*n* = 12 dams), 2–3 h before lights out. The choice of the E8–E18 exposure window was made to match the lab’s previous work on single chemicals to enable direct comparisons [39,40], with the original age range chosen to span a period prior to and during the beginning of the period of brain sexual differentiation [41,42]. Details on the mixture and vehicle are provided below. Treatment groups were randomly assigned and were coded to keep investigators blind to treatment. Dams were provided nestlets beginning on E18.

The offspring (F1 generation) was the current study’s experimental subjects. On the day after birth (postnatal day (P) 1), body weights and anogenital distances (AGD) of pups were measured, and sex ratios were determined. Litters were culled to 5 males and 5 females each, with the animals closest to the median AGD retained. On average, 2 pups per sex per litter were used, with 2–3 pups of each sex assigned to the no-stress arm of the study and 1–3 pups to the stress arm of the study. When the litter was considered as a covariate in a subset of variables, no litter effects were found, in agreement with all of our previous work; therefore, data from individual rats were used as the statistical unit. During early postnatal development, body weight and AGD were measured weekly. Anogenital index (AGI) was calculated as AGD/∛BW. On P21, rats were weaned into same-sex cages, after which body weight was measured weekly. All animals were monitored for the timing of puberty (vaginal opening or preputial separation), and females were subsequently given daily vaginal lavage to monitor estrous cyclicity. After completion of behavioral work at P91, rats were euthanized by rapid decapitation, brains removed, frozen on dry ice, then stored at −80 °C. Females were euthanized on their first proestrus after behaviors were completed. A terminal blood sample was collected, centrifuged, and serum separated, frozen, and stored at −80 °C.

Stimulus rats for the sociability and mate preference behaviors were prepared using adult Sprague Dawley rats brought into the colony (Envigo) and housed under the same conditions as experimental rats. Stimulus males were castrated under isoflurane anesthesia; a subset of males was implanted with a 1.5 cm Silastic capsule containing 100% testosterone during the castration surgery. Stimulus females were ovariectomized under isoflurance anesthesia, with a subset receiving a 1 cm Silastic capsule containing 17β-estradiol (5% estradiol in 95% cholesterol). These rats were between 2 and 3.5 months of age when used in behavioral tests.

### 2.2. NeuroMix Design and Preparation

The NeuroMix was designed to represent common chemicals detectable in humans and associated with neurobiological effects as indicated by the epidemiological and/or experimental animal data discussed above. Dosages were selected to be below the NOAEL in humans. The final concentration of the vehicle was 0.06% DMSO and 0.0037% ethanol in sesame oil. Details of the NeuroMix are shown in Table 1. A single stock solution was produced and used for dosing all of the experimental animals in this study to minimize variability in the number of chemicals received by each dam. This was particularly important for PFOS, which is a surfactant [43] that may not dissolve as well as the other lipophilic chemicals.

Each day from E8 to E18, rat dams were fed ¼ ‘Nilla wafer, which was treated with ~100 μL of the assigned solution. The dams were observed while they consumed the cookie to ensure it was all eaten. The oral route was selected to model human exposures to EDCs, which often occur via the diet. We note the caveat that we did not measure concentrations of chemicals in the dam or transfer to the embryos; however, such transfer is likely to have happened based on robust evidence for transplacental transfer of EDCs, and presence of chemicals in amniotic fluid, fetal tissues, and umbilical cord blood [44,45,46,47]. Appropriate personal protective equipment was used when handling the chemicals. Experimenters were blind to treatment.

**Table 1:** Composition of the NeuroMix.

Chemical	Dose	Vendor	Catalog # (Lot #)	NOAEL
Aroclor 1221 (A1221)	100 µg/kg	Accustandard	C-221N-50MG (072-202-01)	1 mg/kg/d [48]
Bisphenol A (BPA)	2.5 µg/kg	Accustandard	BPA-A-N (11625)	5 mg/kg/d [49]
Bisphenol S (BPS)	2.5 µg/kg	Accustandard	BPA-S-N (20696)	20 mg/kg/d [50]
Di(2-EthylHexyl)-	20 µg/kg	Accustandard	ALR-097N (03312KZ-1-01)	4.8 mg/kg/d [51]
phthalate (DEHP)				
Di-n-butyl phthalate (DBP)	20 µg/kg	Accustandard	ALR-104N (24227)	50 mg/kg/d [52]
Perfluorooctane	20 µg/kg	Accustandard	PFOS-002N (24187)	0.4 mg/kg/d [53]
sulfonate (PFOS)				
Polybrominated diphenyl	100 µg/kg	Sigma	BCBR7972 (91834)	0.7 mg/kg/d [54]
ether 47 (PBDE-47)				
PCB-153	100 µg/kg	Chem Service	BZ-153-10MG (7582000)	16 mg/kg/d [55]
Vinclozolin	100 µg/kg	Chem Service	N-13745-250MG (7416000)	6–12 mg/kg/d [56]

### 2.3. Chronic Restraint Stress

In order to ascertain the interactions of NeuroMix exposure with a postnatal insult (restraint stress during adolescence), a subset of the offspring (1–3 per litter) were randomly assigned to the chronic restraint stress (CRS) arm of the study. From P35 to P44, these rats were subjected to five 90-min sessions given every 2–3 days over 10 days. The sessions were conducted at different times during the dark cycle to add unpredictability to the paradigm. The methods were based on previous restraint stress studies [57]. The restraint was a 25.4 cm^2^ metal mesh sheet folded into a tube. A binder clip was fastened to one end of the tube; after the animal was coaxed into entering the tube, a second binder closed the entry. Rats were not compressed, but they were unable to roll or turn around. Once the rats were in their restraints, they were placed into chambers with their similarly-restrained cage mates and left for a 90-min period. Control (unstressed) littermates were handled but not restrained. The restraints and binders were washed in hot soapy water after each session and left to dry.

### 2.4. Behavioral Testing

Behaviors were tested beginning on P60. The order of testing was the light:dark box, sociability, social novelty, and mate preference, and testing began 1–2 h after lights out. All tests were video recorded, and Stoelting ANY-maze video tracking software was used to analyze movement, distance traveled, position, speed, and interactions with other animals. All behaviors were tested, recorded, and analyzed as per published protocols [6,22]. Final samples sizes for behaviors were: vehicle–unstressed (females: 27, males: 28), vehicle–stress (females: 19, males: 21), NeuroMix–unstressed (females: 30, male: 30), and NeuroMix–stress (females: 22, males: 22).

#### 2.4.1. Light:Dark Box

A rat was placed into a box divided with a partition into 2 chambers (50 × 50 cm each, Stoelting, Wood Dale, IL, USA) as described in [22]. One chamber (“light”) was clear Plexiglas, the other (“dark”) opaque black, with a passageway between. The light chamber was dimly illuminated with a 60 w bulb, and the dark chamber was covered with a lid that allowed red light to pass, enabling observation. For each test, a rat was placed at the passageway facing the light field. Animals were allowed to explore for 5 min, after which they were returned to their home cages.

#### 2.4.2. Sociability and Social Novelty

Five to 10 days after the light:dark box test, each experimental rat was habituated to a three-chambered apparatus (100 × 100 cm; Stoelting), partitioned into three compartments between which were interconnecting doors [5]. A small cylindrical barred cage was located in the far corners of the lateral compartments. After 5 min of exploration of the center chamber, the experimental rat was removed, and a stimulus cage was placed in each of the far corners. For the sociability portion of the test, which was performed first, one cage was empty, and the other contained a same-sex gonadectomized rat. The experimental rat was allowed to explore the entire apparatus for 10 min, then removed to a holding cage. The two stimulus cages were removed. The cage containing the now-familiar rat was randomly placed in one of the far corners. Another cage containing a novel rat (same-sex gonadectomized) was placed in the other corner. For the social novelty part of the test, the experimental rat was returned and allowed to explore for 10 min.

#### 2.4.3. Mate Preference

Mate preference was conducted 5–10 days after the social behavior tests using the same 3-chambered apparatus. One stimulus cage contained an opposite-sex gonadectomized rat that had not been treated with a hormone capsule (“no hormone”). The other stimulus cage contained an opposite-sex, hormone capsule-implanted rat (“hormone”). Stimulus animals were always unfamiliar to the experimental rat. Experimental females were used on the day of proestrus and were confirmed to be receptive by placing with a sexually experienced male and observing for a lordosis response. Stimulus female rats with hormones were implanted with a Silastic capsule of estradiol and given a progesterone injection (600 mg) 2 h before lights out. The experimental rats were allowed to explore the entire apparatus for 10 min.

### 2.5. Brain Preparation, RNA Isolation, and qPCR

Frozen brains were sectioned into 500 μm coronal sections on slides using an NX50 cryostat, and stored at −80 C. Slides were subsequently placed on a freezing stage (−18 °C), and regions of interest were bilaterally punched from the coronal sections using a Palkovits punch (Stoelting, Wood Dale, IL, USA) (0.75 mm diameter), placed in chilled microfuge tubes, and stored at −80 °C [6]. The medial amygdala (MeA) and paraventricular nucleus (PVN) were selected as regions of interest for qPCR. An in-house cushion/lysis buffer was used for RNA isolation and purification, applied to a spin column (Epoch Life Science, Missouri City, Tx DNA/RNA Spin column; [58]) centrifuged for 30 s at 13,000× *g*. The RNA retained in the column was eluted, quantity determined by a nanodrop, and diluted for cDNA conversion (High Capacity cDNA Reverse Transcription Kit, Thermo Fisher Scientific, Carlsbad, CA, USA). Real-time PCR was run using TaqMan Gene Expression Master Mix (Thermo Fisher Scientific) on a ViiA7 Real-time PCR System (Applied Biosystems, Carlsbad, CA, USA) at 50 °C (2 min), 95 °C (10 min), and 45 cycles of 95 °C (15 s) and 60 °C (1 min). Primers and probes were purchased from Thermo Fisher Scientific for androgen receptor (*Ar*) and estrogen receptor beta (*Esr2*), duplexed to *Gapdh*. Relative expression in each sample was determined using the comparative Ct method [59,60]. Samples were normalized to *Gapdh* and calibrated to the median δ-cycle threshold of the unstressed vehicle females.

### 2.6. Corticosterone RIA

Serum collected at euthanasia was run in duplicate samples (10 μL per sample) in a subset of 12 rats per group. All samples were run in a single corticosterone RIA (MP Biomedicals, Solon, OH, USA, Cat. #07120102). Assay sensitivity was 7.7 ng/mL, and intra-assay variability was 1.92%.

### 2.7. Statistical Analysis

In order to establish sexually dimorphic profiles of developmental, hormonal, behavioral, and gene expression data, results for all females and all males (treatment and stress collapsed) were compared by *t*-test. Effect sizes were determined by Cohen’s d, in which the large effect size was 0.8, and the medium effect size was 0.5. Subsequently, because of large sex differences in many of the variables, data were analyzed separately within each sex. Longitudinal data on body weight and anogenital distance or anogenital index were analyzed by two-way repeated-measured ANOVA. Body weight data were subdivided into 3 age categories due to differences in body weight trajectories. Other endpoints were analyzed by two-way ANOVA or comparable non-parametric if data did not meet the criteria for parametric analysis. Effect sizes for two-way analyses were calculated for ANOVAs by partial eta-squared, with a large effect size of 0.14 and a medium effect size of 0.06. For Kruskal–Wallis non-parametric, effect sizes were determined by epsilon squared, with a large effect size of 0.26 and a medium effect at 0.08. Variables within each sex were treatment (NeuroMix vs. vehicle) and stress (CRS vs. no stress). *Post hoc* analysis was conducted using Tukey’s (parametric) or Bonferroni (non-parametric) tests.

## 3. Results

A summary of detailed statistics for the complete study is provided in Table S1. In order to determine sex differences in these endpoints, differences by sex in each measure were calculated with data collapsed across treatment and stress. The results shown in Table 2 confirmed the expected dimorphisms in numerous endpoints. Subsequent analyses of the effects of NeuroMix and stress were performed within the sexes.

### 3.1. Development

#### 3.1.1. Body Weight

Longitudinal body weight data were measured throughout life and analyzed separately by repeated-measures ANOVA through prepuberty, adolescence, and adulthood because of differences in the body weight trajectories across postnatal development.

**Figure 1:**
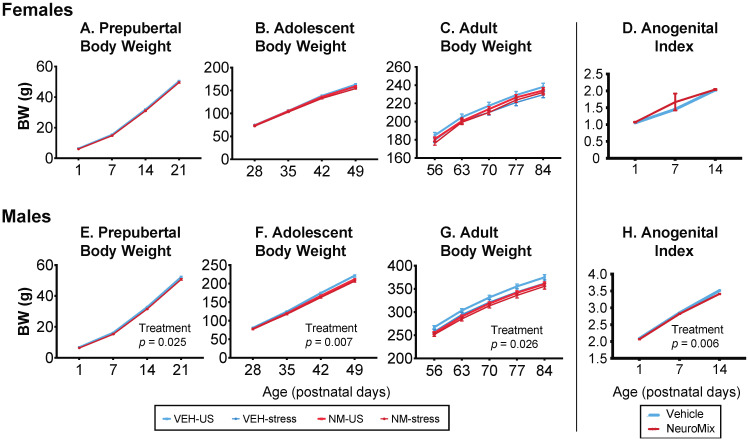
Effects of prenatal NeuroMix and/or stress in adolescence on body weight in females (**A**–**C**) and males (**E**–**G**) across prepubertal, adolescent, and adult life stages. In females, there were no effects of treatment or stress. In males, repeated measures ANOVA found that body weight was significantly affected by treatment through each of the three life stages. Specifically, vehicle–unstressed rats had higher body weights than the other groups; *p*-values for the main effects of treatment are indicated in panels (**E**–**G**). Anogenital index (AGI) in prepubertal rats was unaffected in females (**D**) and was significantly decreased by NeuroMix in males, with the *p*-value for the significant main treatment effect shown (**H**). Prepubertal females: *n* = 46 vehicle and 52 NeuroMix; prepubertal males: *n* = 49 Vehicle, *n* = 52 NeuroMix. For vehicle–unstressed, vehicle–stressed, NeuroMix–unstressed, and NeuroMix–stressed adolescent and adult females, *n* = 27, 19, 30 and 22; for males, *n* = 28, 21, 30, and 22.

Females: In females, there were no significant effects of NeuroMix, stress, or their interactions at any of the three developmental periods (Figure 1A–C).

Males: Prior to puberty, body weight was decreased by NeuroMix (*p* = 0.025; Figure 1E). In adolescence, during which restraint stress was given to a subset of rats from P35 to P44, NeuroMix treatment (*p* = 0.007) decreased body weight, and there was a trend for stress to decrease body weight (*p* = 0.055; Figure 1F). Similar results were found in adulthood (treatment: *p* = 0.026; stress: *p* = 0.097; Figure 1G). There were no interactions of treatment and stress.

#### 3.1.2. Anogenital Index (AGI)

The anogenital distance was measured on P1, 7, and 14. The anogenital index was calculated as AGD/∛BW.

Females: Females were unaffected by NeuroMix (*p* = 0.347; Figure 1D).

Males: In males, AGI was significantly smaller in NeuroMix than vehicle (*p* = 0.006; Figure 1H).

#### 3.1.3. Puberty

Puberty was monitored to determine the effects of NeuroMix, and the subsequent influence of stress given in adolescence. We note that the age range for chronic restraint stress was from P35 to P44, which overlaps with when vaginal opening in females and preputial separation in males normally occurs in our colony. Therefore the interpretation of these data should keep this overlap in mind.

**Figure 2:**
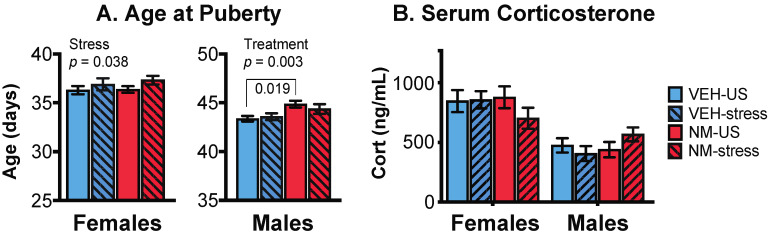
Effects of prenatal NeuroMix and/or stress in adolescence on the timing of puberty in female (A) and male (B) rats, and on serum corticosterone concentrations (C). (**A**) In females, the age at vaginal opening was significantly delayed by stress (striped bars). In males, treatment significantly delayed the timing of puberty, and there was also an interaction of treatment and stress as indicated by the brackets. Sample sizes for puberty are the same as in Figure 1. (**B**) Serum corticosterone concentrations were unaffected by treatment or adolescent stress. Sample sizes for serum corticosterone are *n* = 12 per group. Cort = corticosterone; VEH = Vehicle; NM = NeuroMix; US = unstressed.

Females: Puberty in females was assessed by the day of the vaginal opening (VO). There was no main effect of NeuroMix (*p* = 0.352), but a significant effect of stress, with the stressed females having a significant delay of VO compared to unstressed females (*p* = 0.038; Figure 2A, left). No interaction of treatment and stress was found (*p* = 0.155).

Males: In males, the timing of puberty was determined by the day of preputial separation (PPS). There was a significant main effect of NeuroMix on male puberty (*p* = 0.003), which was delayed in NeuroMix compared to vehicle. There was no effect of stress (*p* = 0.626), but there was a significant interaction of treatment and stress (*p* = 0.017; Figure 2A, right), the latter attributable to the NeuroMix–stressed males having significantly later puberty than the vehicle–stressed males (*p* = 0.019).

### 3.2. Serum Corticosterone

Serum corticosterone was assayed in samples collected at euthanasia, with females in proestrus. The expected sex difference (female > male) was found (*p* < 0.0001), but there were no effects of treatment, stress, or their interactions, on corticosterone concentrations in either sex (Figure 2B).

### 3.3. Behaviors

All rats were run through a behavioral battery beginning in adulthood (~P60) in the same order (light:dark box, sociability, social novelty, and mate preference).

#### 3.3.1. Light:Dark Box (LD)

**Figure 3:**
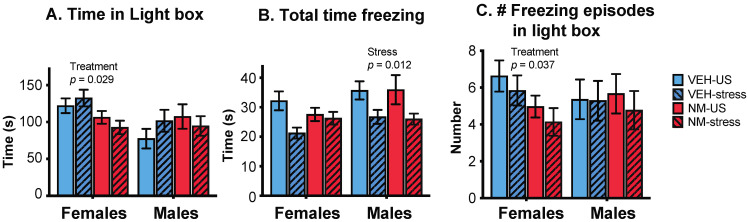
Effects of prenatal NeuroMix and/or stress in adolescence on behaviors in the light:dark box. (**A**) Time spent in the light box was significantly decreased in NeuroMix compared to vehicle in females. (**B**) Total time freezing was decreased by adolescent stress in males, and there was also a trend for a decrease in stress in females (*p* = 0.066). (**C**) The number of freezing episodes in the light box was significantly decreased by treatment in females and unaffected in males. Sample sizes for vehicle–US, vehicle–stress, NeuroMix–US, and NeuroMix–stressed females: *n* = 27, 19, 30 and 22; for males, *n* = 28, 21, 30, and 22. VEH = Vehicle; NM = NeuroMix; US = unstressed.

Females: There was a significant effect of treatment, with NeuroMix females spending less time in the light box than vehicle females (*p* = 0.029; Figure 3A) and correspondingly more time in the dark box (data not shown). Total time spent freezing was unaffected in females, although there was a trend for stress to decrease freezing (*p* = 0.066; Figure 3B). The number of freezing episodes in the light box was decreased in NeuroMix females (*p* = 0.037; Figure 3C). Time spent freezing in the light box was unaffected (data not shown).

Males: There were no main effects of treatment on the behavior of males in the LD box, but there were main effects of stress. Males that experienced stress during adolescence spent less total time freezing (*p* = 0.012; Figure 3B) than non-stressed males; this was similar in the light and the dark compartments (not shown). There were no effects of stress on overall time spent in either compartment (time in the light box shown in Figure 3A), and there were no differences in the number of freezing episodes in any compartments (shown for the light box in Figure 3C).

#### 3.3.2. Sociability

**Figure 4:**
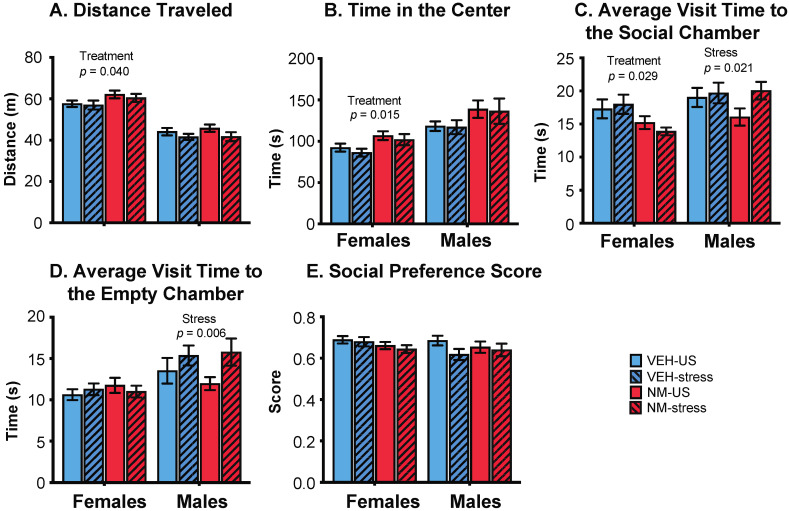
Effects of prenatal NeuroMix and/or stress in adolescence on behaviors in the sociability test in which rats were offered a choice between a same-sex conspecific vs. an empty cage. (**A**) Distance traveled and (**B**) time spent in the center chamber were increased by NeuroMix compared to vehicle in females. (**C**) Average time spent per visit to the social chamber was decreased by NeuroMix treatment in females, and increased by stress in males. (**D**) Average time spent per visit to the empty chamber was increased by stress in males. (**E**) Social preference score was unaffected. Sample sizes for vehicle–unstressed, vehicle–stress, NeuroMix–unstressed, and NeuroMix–stressed females: *n* = 27, 19, 30 and 22; for males, *n* = 28, 20, 29, and 22. VEH = vehicle; NM = NeuroMix; US = unstressed.

Females: In the sociability task, females treated with NeuroMix traveled farther and faster than vehicle females (*p* = 0.040, 0.038, respectively; Figure 4A). There was no effect of stress nor an interaction of stress and treatment on these endpoints. NeuroMix females also spent more time in the center chamber relative to vehicle females (*p* = 0.015; Figure 4B). Time spent near the social chamber was unaffected by treatment or stress, although a trend for a decrease with treatment was found (*p* = 0.059; data not shown). The average amount of time spent per visit to the social chamber was decreased by treatment in females (*p* = 0.029; Figure 4C). The social preference score, calculated as time spent near the social animal divided by the total time spent near the social animal and the empty chamber, was not affected in either sex (Figure 4E).

Males: No endpoints in the sociability task were affected by NeuroMix treatment, but there were stress effects. The average amount of time spent per visit to the social chamber was increased by stress in males (*p* = 0.021; Figure 4C). Similarly, the average amount of time spent per visit to the empty chamber was increased by stress in males (*p* = 0.006; Figure 4D). The social preference score was unaffected (Figure 4E).

#### 3.3.3. Social Novelty

**Figure 5:**
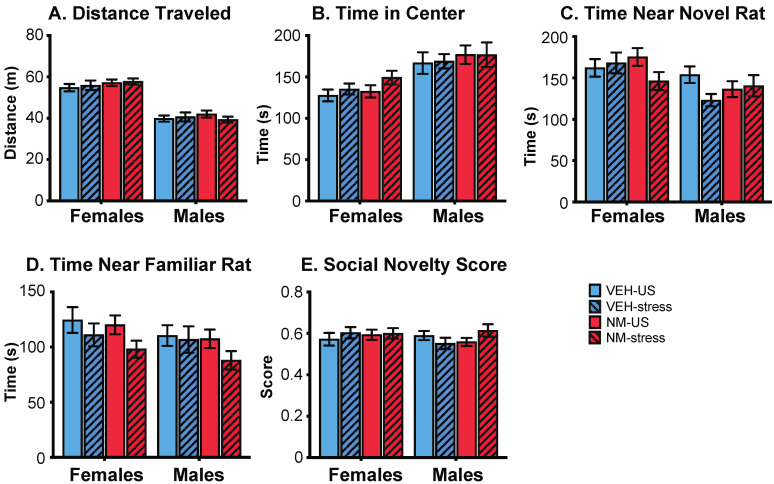
Effects of prenatal NeuroMix and/or stress in adolescence on behaviors in the Social Novelty test in which rats were offered a choice between a familiar and a novel conspecific. No significant effects were found on (**A**) distance traveled, (**B**) time in center, (**C**) time near the novel rat, (**D**) time near the familiar rat, and (**E**) social novelty score. Sample sizes are the same as in Figure 4. VEH = vehicle; NM = NeuroMix; US = unstressed.

There were no significant treatment or stress effects, nor any interactions, found in either sex in the social novelty task (Figure 5).

#### 3.3.4. Mate Preference

**Figure 6:**
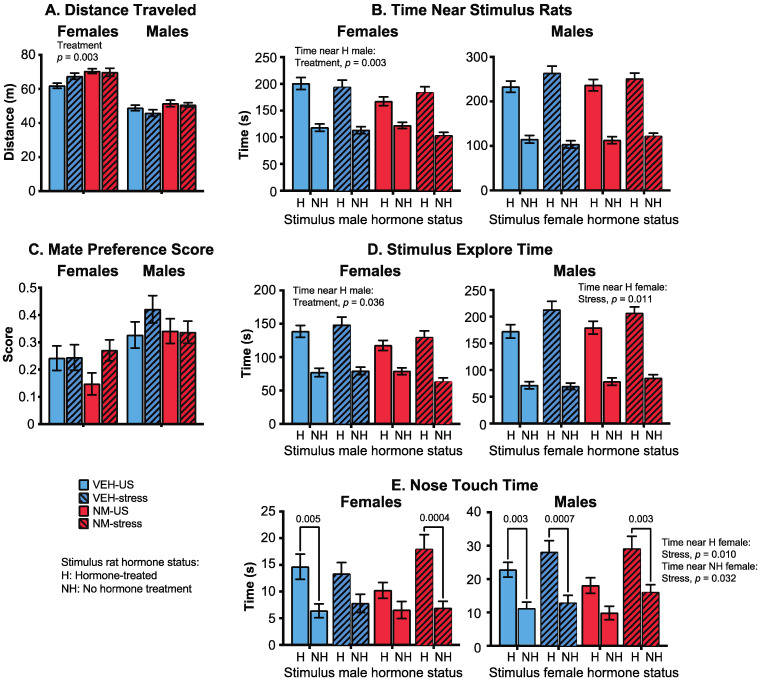
Effects of prenatal NeuroMix and/or stress in adolescence on behaviors in the Mate preference test in which rats were offered a choice between two opposite-sex conspecifics, both castrated, with one hormone-treated (H) and the other not given hormone (NH). (**A**) Distance traveled was increased by treatment in females. (**B**) Time spent near the stimulus rats was affected only in females, with time spent near the hormone-treated male significantly decreased by NeuroMix compared to vehicle. (**C**) Mate preference score was unaffected. (**D**) Stimulus explore time, defined as time spent engaging with the stimulus rat, was affected in females, with time exploring near the hormone-treated male decreased by NeuroMix compared to vehicle. In males, stress increased stimulus explore time spent near the hormone-treated stimulus female. (**E**) Time spent nose touching was affected in both sexes. Females in the vehicle–unstressed and NeuroMix–Stressed groups, showed the expected preference for a hormone over a no-hormone male, an effect that was not seen in the vehicle–stress or NeuroMix–unstressed females. In males, the expected preference for hormone- over no-hormone-treated females was found in all groups except the NeuroMix–unstressed group. In addition, in males, stress increased time near both the hormone- and no-hormone-treated females. Sample sizes for vehicle–unstressed, vehicle–stress, NeuroMix–unstressed, and NeuroMix–stressed females: *n* = 27, 19, 28 and 22; for males, *n* = 28, 20, 30, and 22. VEH = vehicle; NM = NeuroMix; US = unstressed.

Females: Distance traveled by females was increased in the NeuroMix females compared to vehicle (*p* = 0.003; Figure 6A) with no main effect of stress. In general, time spent near or interacting with the stimulus animals was decreased by NeuroMix treatment in females. This was significant for time spent near the hormone-replaced male (*p* = 0.049; Figure 6B), and total time spent with both stimulus males together (*p* = 0.025; data not shown). The mate preference score, calculated as [time near hormone–time near non-hormone]/total social time, was not affected (Figure 6C). The stimulus explore time with the hormone-replaced male was also decreased by NeuroMix relative to vehicle (*p* = 0.036; Figure 6D), whereas the time with the no hormone male was unaffected. Analysis of time females spent nose touching with the stimulus males revealed that the expected preference for a hormone over a no-hormone male was observed in vehicle–unstressed females and NeuroMix–stressed females (*p* = 0.005, 0.0004, respectively), a preference that was abolished in the vehicle–stressed and the NeuroMix–unstressed females (Figure 6E).

Males: There were no effects of treatment or stress on distance traveled (Figure 6A). Time near the stimulus females was unaffected, with all of the male groups showing the expected preference for a hormone-treated over a non-hormone-treated female (Figure 6B). The mate preference score was similarly unaffected (Figure 6C). Stimulus explore time was unaffected by treatment, but the stress caused a significant increase in time spent by males with the hormone-treated stimulus females (*p* = 0.011; Figure 6D). Finally, nose touch time was significantly affected by treatment: all groups except the NeuroMix–unstressed males showed the expected preference for a hormone over a non-hormone treated stimulus female (Figure 6E). There was also a significant stress effect in nose touch time with both the hormone-treated (*p* = 0.010) and the no-hormone-treated female (*p* = 0.032), both of which were increased by stress.

### 3.4. Gene Expression

**Figure 7:**
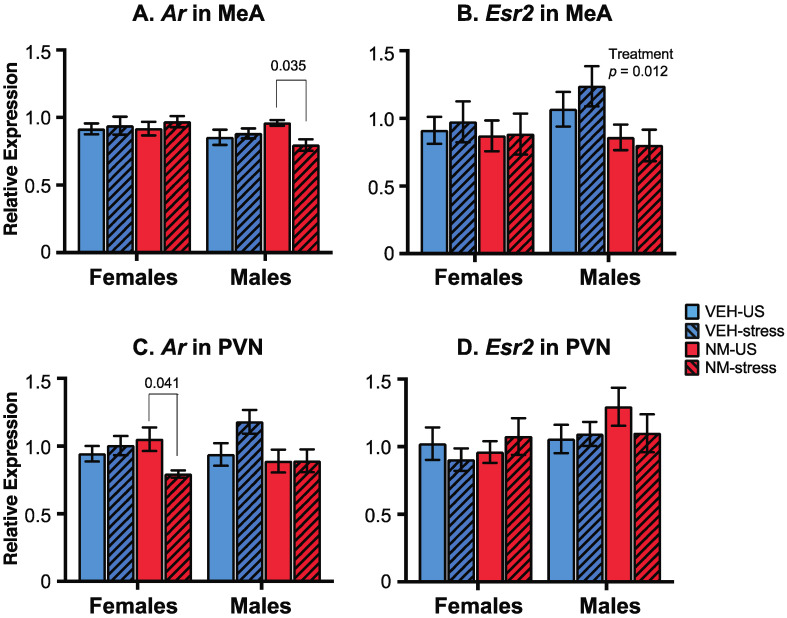
Effects of prenatal NeuroMix and/or stress in adolescence on gene expression in the medial amygdala (MeA) and the paraventricular nucleus (PVN). (**A**) For *Ar* in the MeA, a significant interaction of stress and treatment was found in males. (**B**) In the MeA, *Esr2* was significantly decreased by NeuroMix in males. (**C**) *Ar* in the PVN had an interaction of treatment and sex in females. (**D**) *Esr2* was unaffected in the PVN. *n* = 11–12 per group. VEH = vehicle; NM = NeuroMix; US = unstressed.

The expression of androgen receptor (*Ar*) and estrogen receptor β (*Esr2*) genes was measured by qPCR in two post-mortem brain regions of the experimental rats, with regions chosen based on their roles in the behaviors studied. In the medial amygdala (MeA), *Ar* was unaffected in females. In males, there were no main effects of treatment or stress, but there was a significant interaction (*p* = 0.048; Figure 7A). *Post hoc* analysis showed that the NeuroMix–unstressed males had higher *Ar* compared to NeuroMix–stressed (*p* = 0.035). For *Esr2* in the MeA, there were no effects in females, but males were significantly affected by treatment (*p* = 0.012; Figure 7B) with *Esr2* expression in NeuroMix lower than vehicle males. No main effects of stress or interactions were detected.

In the paraventricular nucleus (PVN), while *Ar* was unaffected by treatment (*p* = 0.093) or stress (*p* = 0.144) in females, a significant interaction was found (*p* = 0.022; Figure 7C) attributable to the NeuroMix–unstressed females having higher *Ar* expression relative to NeuroMix–stressed females (*p* = 0.041). In males, *Ar* was not significantly affected by treatment, although a trend for decreased expression in NeuroMix was found (*p* = 0.053; Figure 7C). Neither stress nor an interaction was found for *Ar*. *Esr2* expression in the PVN was unaffected (Figure 7D).

**Figure 8:**
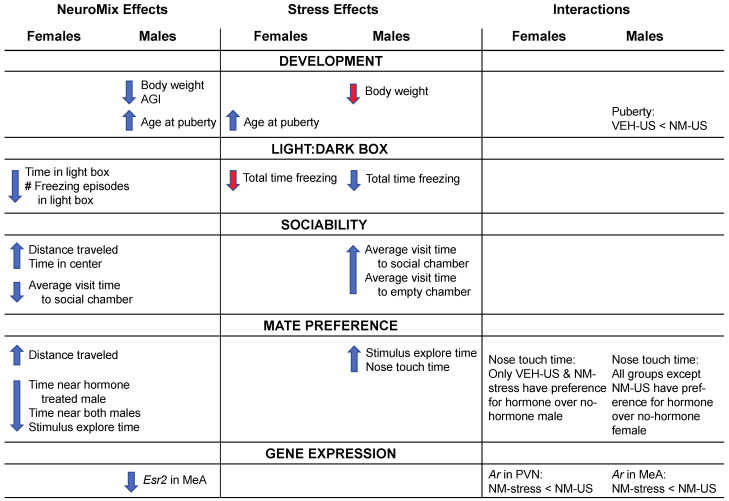
Schematic of overall outcomes of prenatal NeuroMix exposure, stress during adolescence, and their interactions. Down-arrows indicate decreases caused by NeuroMix vs. vehicle or stress vs. no stress. Up-arrows indicated increases caused by NeuroMix vs. vehicle or stress vs. no stress. Significant differences (at *p* < 0.05) are indicated by blue arrows, and trends (0.05 < *p* < 0.1) are indicated by red arrows. US: unstressed; NM: NeuroMix; VEH: vehicle; PVN: paraventricular nucleus; MeA: medial amygdala; AGI: anogenital index.

Figure 8 provides a schematic summary of overall findings for effects of NeuroMix, stress, and their interactions.

## 4. Discussion

A growing body of evidence from animal studies, human epidemiology, and biomonitoring has demonstrated that environmental chemical mixtures represent the real-world situation in wildlife and humans and that chemicals in combination may have effects that may not be predictable from single compounds [23,26,27,30,61,62]. In the current study, we designed a mixture comprising bisphenols, phthalates, perfluorinated, polybrominated and polychlorinated compounds, and vinclozolin. Their selection for the NeuroMix was based on evidence showing that each chemical individually causes deficits in behaviors and/or changes in neurobiological markers in animals [5,6,19,63,64,65,66] and is detectable in humans [30,32,67,68,69,70,71,72]. This current study adds to the literature by using these chemicals in combination at human-relevant dosages and administered by the most relevant (oral) route.

The second factor of this study was how the NeuroMix might interact with other environmental stressors. We are all subjected to other pollutants, temperature extremes, psychosocial stress, pathogens, nutritional stress, and others. Here, we decided to combine our prenatal NeuroMix exposure with postnatal stress in adolescence. This choice was made because stress experiences can lead to neurodevelopmental and behavioral disorders [73,74]. In addition, we previously used restraint stress as a model for studying interactions of EDCs and stress in adolescence across generations [57], identifying what we referred to as a “synchronicity” amongst ancestral exposure and proximate stress on behavioral and neuromolecular outcomes.

As a whole, the results revealed sex differences in responses to prenatal NeuroMix exposure and to stress in adolescence. In females, NeuroMix altered numerous behaviors tested, whereas stress delayed puberty but did not affect other endpoints. In males, EDC treatments affected development (delayed puberty, decreased body weight, and anogenital index) but had no effects on behaviors. Instead, males were sensitive to adolescent stress, with a number of behavioral changes observed. Interactions of NeuroMix and stress were observed for just a few endpoints, namely, the timing of puberty, mate preference behavior, and expression of the *Ar* gene in the brain. These results are discussed below in the context of other findings on EDC and stress actions on development and behavior.

### 4.1. EDC and Stress Effects on Postnatal Development

The EDCs used in this study act through different hormonal pathways, including those mediated by nuclear hormone receptors such as estrogen receptors (e.g., PCBs, BPA, BPS; [75,76]), androgen receptors (e.g., vinclozolin, phthalates; [77,78]), PPARγ (perfluorinated compounds; [79]), and aryl hydrocarbon receptors (PCB-153, PBDE-47; [80,81]), among others. Perfluorinated chemicals such as PFOS likely act through other families of receptors, with actions mediated by both nuclear (e.g., PPARs: [82]; ERα: [83]) and membrane receptors, including non-genomic estrogen receptors [84] and GPR40 [85]. This complexity means that effects of the mixture may be mediated by different combinations of receptors in a tissue- and sex-specific manner depending upon abundance and distribution, along with differential developmental sensitivities.

Developmental effects of NeuroMix were limited to males, which exhibited delayed timing of puberty and decreased anogenital index and body weight. The delayed sexual maturation in the males is consistent with a previous study [78] that showed that developmental exposure to anti-androgens can lead to delayed preputial separation. The effects of various EDCs on body weight were also reported, although the majority of studies have suggested obesogenic effects of EDCs [86]. Nevertheless, some studies in rodents and humans have demonstrated decreased body weight in association with BPA and BPS in rats [87] and PCBs and PBDEs in humans [88,89]. Decreases in the anogenital distance in males, as in the current study, are consistent with the effects of anti-androgenic EDCs such as vinclozolin [90].

It is interesting that females were resistant to NeuroMix effects on development, with no change in body weight or puberty. The lack of effect on the timing of puberty was surprising, as the mixture contains estrogenic EDCs previously shown to advance the timing of puberty [91,92]. It is likely that the presence of EDCs with anti-androgenic effects or other hormonal mechanisms that interact with the effects of estrogens may have counteracted these actions. By contrast, restraint stress in adolescence delayed the timing of puberty in females. The literature on stress and puberty is inconsistent, with examples of accelerated [93,94], delayed [95,96], or no change [97] in the timing of puberty: differences likely attributable to factors including genetic susceptibility, species differences, the timing of exposure, and others.

### 4.2. EDC and Stress Effects on Behaviors

In this study, we made the surprising observation that NeuroMix influenced a broad range of behaviors in female rats but had no effect in males, with the exception of the mate preference test. This greater female sensitivity is in direct contrast to the developmental effects of NeuroMix discussed above, in which males had a greater vulnerability. More specifically, in the light:dark box, which is a test of anxiety-like behaviors, NeuroMix females spent less time in the light box, and while they were not significantly different from vehicle females in time freezing, the NeuroMix females had fewer freezing episodes. These results of altered anxiety-like behaviors are consistent with other behavioral work on developmental EDCs [17,20,21,98,99]. In a test of sociability, in which a rat is given a choice between a same-sex conspecific or an empty cage, NeuroMix females showed decreased sociability and increased locomotor activity. We did not find any effects of treatment in the social novelty test. Other studies on EDCs have also revealed deficits in aspects of social behaviors ([99,100]; reviewed in [1]).

In the anxiety and social behaviors, effects of restraint stress in adolescence were limited to male rats, in which stress decreased time freezing in the light:dark box, and increased time spent in proximity to both the conspecific chamber and the empty chamber in the sociability test. These latter results do not point to any wholesale disruption of anxiety or sociability in males but rather suggest that specific aspects of behaviors are perturbed by stress. Our finding of greater male vulnerability is surprising in light of other evidence that in mice, females are more vulnerable [101,102]. However, there may be species differences, as in rats, adolescent stress can have greater, lesser, or similar effects in males compared to females depending upon the model [103,104,105].

The most interesting behavioral results were in the mate preference test. Again, females were mainly influenced by prenatal NeuroMix, and males by adolescent stress, but there were also interactions of these two factors. In females, time spent with the stimulus males was decreased by NeuroMix. For nose touching, which we consider to be the most sensitive and nuanced index of interest and investigation between rats [5,6], the preference of females for a hormone over a no-hormone male was decreased by NeuroMix and stress independently. In males, stress (but not NeuroMix) increased time spent with females; an interaction of NeuroMix and stress was found in which the NeuroMix–unstressed males showed no preference for a hormone over a no-hormone female, contrary to the other three groups. Together, these results show that the two stressors interacted in a manner that was dissimilar to the effects of each alone, presumably due to multiple and complex mechanisms that we previously referred to as ‘synchronicity’ [57]. This result is also comparable to our previous two-hit work in which rats were exposed to PCBs during perinatal development, as juveniles, or both [21,35], and wherein single hits at either age did not predict effects of their combination. Our findings are also consistent with the numerous studies suggesting that mate preference is impaired by developmental EDC exposures [6,99,106,107]. Collectively, we believe that this body of work is important because the vulnerability of mate preference and sexual selection more broadly to EDCs can have consequences on the reproduction and fitness of a species [108].

### 4.3. EDC and Stress Effects on Gene Expression

Finally, we analyzed the expression of two steroid hormone receptors involved in mediating EDC effects: the androgen receptor (*Ar*) and estrogen receptor β (*Esr2*). Using brains of behaviorally-characterized rats, we selected the medial amygdala for its role in emotional and anxiety behaviors and based on evidence for effects of EDCs on its morphology and expression of hormone-sensitive genes [109,110,111,112]. We selected the paraventricular nucleus (PVN) for its sensitivity to EDCs, regulation of the hypothalamic–pituitary–gonadal and hypothalamic–pituitary–adrenal axes, and because it plays key roles in social behavior through the release of the nonapeptides, vasopressin, and oxytocin [1,58,113,114]. In the medial amygdala, *Ar* and *Esr2* expression were affected in males but not females, with *Esr2* decreased in NeuroMix males irrespective of stress, and *Ar* decreased in NeuroMix–stressed compared to NeuroMix–unstressed males. In the PVN, *Esr2* was unaffected in either sex, whereas in females, *Ar* was decreased in NeuroMix–stressed compared to NeuroMix–unstressed animals. These interaction effects for *Ar* in the medial amygdala (males) and PVN (females) are among very few interactions found in the current study. We were unable to analyze more genes due to limited quantities of RNA from our samples, but the current results suggest that future studies should include neuromolecular profiling of more genes and additional brain regions.

## 5. Conclusions

Studies on low-dose mixtures of EDCs represent a new frontier in the field as they reveal how combinations of these chemicals may allow them to cause adverse effects below their NOAEL. Chronic stress can cause perturbations in normal processes, which can also lead to adverse effects if it occurs during critical developmental periods. The novelty of this research lies in its investigation of the interaction of NeuroMix and chronic stress, which in previous work was termed synchronicity [57]. The main conclusions to be drawn from this study are that stress in adolescence has the ability to modulate the effects of prenatal EDC exposure in some measures and that most of the neurobehavioral effects of the NeuroMix are specific to females.

## Figures and Tables

**Table 2:** Sex differences.

	Females		Males				
Development and Physiology	Mean	SEM	Mean	SEM	*p* Value	Cohen’s d	Effect Size
P1 Body Weight	6.20	0.07	6.55	0.07	0.120	−0.50	Medium
P84 Body Weight	239	1.7	383	3.4	*<0.0001*	−5.42	Large
P1 AGI	1.06	0.01	2.08	0.01	*<0.0001*	−11.21	Large
P14 AGI	2.03	0.01	3.46	0.01	*<0.0001*	−12.09	Large
Corticosterone	821	43	472	31	*<0.0001*	1.34	Large
**Light/Dark**	**Mean**	**SEM**	**Mean**	**SEM**	***p* value**	**Cohen’s d**	**Effect Size**
Distance	24.35	0.41	19.76	0.40	*<0.0001*	1.13	Large
Freezing Episodes	22.28	0.93	23.59	1.07	0.283	−0.13	Small
Time Freezing	27.31	1.31	31.78	1.85	*0.050*	−0.28	Small
Light Time	113	5.0	95	7.5	*0.040*	0.28	Small
Light Freezing Episodes	5.41	0.38	5.31	0.53	0.854	0.02	Small
Light Time Freezing	6.77	0.69	7.65	1.02	0.479	−0.10	Small
Dark Time	186	5.0	203	7.5	*0.045*	−0.27	Small
Dark Freezing Episodes	16.79	0.93	18.06	1.17	0.324	−0.12	Small
Dark Time Freezing	20.28	1.21	23.61	1.80	0.115	−0.22	Small
Line Crossing	16.66	0.59	11.50	0.79	*<0.0001*	0.74	Medium
**Sociability**	**Mean**	**SEM**	**Mean**	**SEM**	***p* value**	**Cohen’s d**	**Effect Size**
Distance	59.44	0.95	43.57	0.93	*<0.001*	1.71	Large
Time In Center	97.52	2.82	128.16	5.22	*<0.001*	−0.73	Medium
Time Near Social	230	5.0	208	6.5	*0.002*	0.39	Small
Avg Visit Time to Social	16.05	0.60	18.49	0.71	*0.016*	−0.38	Small
Time Near Empty	113	3.6	108	4.5	0.440	0.14	Small
Avg Visit Time to Empty	11.17	0.39	13.94	0.67	*<0.001*	−0.51	Large
Social Pref Score	0.67	0.01	0.65	0.01	0.255	0.14	Small
**Social Novelty**	**Mean**	**SEM**	**Mean**	**SEM**	***p* value**	**Cohen’s d**	**Effect Size**
Distance	56.38	0.89	40.53	0.84	*<0.001*	1.85	Large
Time in Center	136	3.8	172	6.1	*<0.001*	−0.73	Medium
Time Near Familiar	115	5.0	104	4.7	0.123	0.22	Small
Avg Visit Time Familiar	10.81	0.49	12.96	0.77	*0.020*	−0.34	Small
Time Near Novel	164	5.6	140	5.2	*0.002*	0.45	Small
Avg Visit Time Novel	12.44	0.52	14.11	0.58	*0.034*	−0.31	Small
Social Novelty Score	0.59	0.01	0.58	0.01	0.466	0.10	Small
**Mate Preference**	**Mean**	**SEM**	**Mean**	**SEM**	***p* value**	**Cohen’s d**	**Effect Size**
Distance	67.36	0.92	49.42	0.88	*<0.001*	2.00	Large
Time Near Hormone	185	5.5	244	6.7	*<0.001*	−0.97	Large
Time Near NH	115	3.2	113	4.0	0.751	0.05	Small
Stim Explore Time Hormone	132	4.7	190	6.6	*<0.001*	−1.03	Large
Nose Touch Number Hormone	11.52	0.73	17.76	1.01	*<0.001*	−0.71	Medium
Nose Touch Time Hormone	13.77	1.10	23.86	1.48	*<0.001*	−0.78	Medium
Stim Explore Time NH	74.98	2.93	76.10	3.39	0.802	−0.04	Small
Nose Touch Number NH	6.00	0.53	9.54	0.72	*<0.001*	−0.56	Medium
Nose Touch Time NH	6.81	0.74	12.18	1.07	*<0.001*	−0.59	Medium
Time Near Both Rats	300	5.4	358	5.8	*<0.001*	−1.02	Large
Mate Pref Score	0.22	0.02	0.35	0.02	*<0.0001*	−0.59	Medium
**Gene Expression**	**Mean**	**SEM**	**Mean**	**SEM**	***p* value**	**Cohen’s d**	**Effect Size**
MeA *Ar*	0.94	0.02	0.87	0.02	0.062	0.40	Small
MeA *Esr2*	0.91	0.06	0.99	0.06	0.374	−0.18	Small
PVN *Ar*	0.95	0.03	0.97	0.04	0.627	−0.08	Small
PVN *Esr2*	0.99	0.05	1.14	0.06	0.077	−0.37	Small

Note: Significant differences with *p*-values ≤ 0.05 are italicized. Avg: average; NH: no-hormone; Pref: preference; Stim: stimulus; MeA: medial amygdala; PVN: paraventricular nucleus.

## Data Availability

Data will be made available upon request.

## Supplementary Materials

The following are available online at https://www.mdpi.com/article/10.3390/toxics10030122/s1, Table S1: Statistical analyses.

## References

[1] Gore A.C., Krishnan K., Reilly M.P. (2019). Endocrine-disrupting chemicals: Effects on neuroendocrine systems and the neurobiology of social behavior. Horm. Behav..

[2] Sullivan A.W., Beach E.C., Stetzik L.A., Perry A., D’Addezio A.S., Cushing B.S., Patisaul H.B. (2014). A novel model for neuroendocrine toxicology: Neurobehavioral effects of BPA exposure in a prosocial species, the prairie vole (*Microtus ochrogaster*). Endocrinology.

[3] Rosenfeld C.S. (2015). Bisphenol A and phthalate endocrine disruption of parental and social behaviors. Front. Neurosci..

[4] Reilly M.P., Weeks C.D., Crews D., Gore A.C. (2018). Application of a Novel Social Choice Paradigm to Assess Effects of Prenatal Endocrine-Disrupting Chemical Exposure in Rats (*Rattus norvegicus*). J. Comp. Psychol..

[5] Reilly M.P., Weeks C.D., Topper V.Y., Thompson L.M., Crews D., Gore A.C. (2015). The effects of prenatal PCBs on adult social behavior in rats. Horm. Behav..

[6] Topper V.Y., Reilly M.P., Wagner L.M., Thompson L.M., Gillette R., Crews D., Gore A.C. (2019). Social and neuromolecular phenotypes are programmed by prenatal exposures to endocrine-disrupting chemicals. Mol. Cell Endocrinol..

[7] Jones B.A., Watson N.V. (2012). Perinatal BPA exposure demasculinizes males in measures of affect but has no effect on water maze learning in adulthood. Horm. Behav..

[8] Tian Y.H., Baek J.H., Lee S.Y., Jang C.G. (2010). Prenatal and postnatal exposure to bisphenol a induces anxiolytic behaviors and cognitive deficits in mice. Synapse.

[9] Mhaouty-Kodja S., Belzunces L.P., Canivenc M.C., Schroeder H., Chevrier C., Pasquier E. (2018). Impairment of learning and memory performances induced by BPA: Evidences from the literature of a MoA mediated through an ED. Mol. Cell Endocrinol..

[10] Kobrosly R.W., Evans S., Miodovnik A., Barrett E.S., Thurston S.W., Calafat A.M., Swan S.H. (2014). Prenatal phthalate exposures and neurobehavioral development scores in boys and girls at 6–10 years of age. Environ. Health Perspect..

[11] England-Mason G., Martin J.W., MacDonald A., Kinniburgh D., Giesbrecht G.F., Letourneau N., Dewey D. (2020). Similar names, different results: Consistency of the associations between prenatal exposure to phthalates and parent-ratings of behavior problems in preschool children. Environ. Int..

[12] Yoo S.J., Joo H., Kim D., Lim M.H., Kim E., Ha M., Kwon H.J., Paik K.C., Kim K.M. (2020). Associations between Exposure to Bisphenol A and Behavioral and Cognitive Function in Children with Attention-deficit/Hyperactivity Disorder: A Case-control Study. Clin. Psychopharmacol. Neurosci..

[13] Perera F., Nolte E.L.R., Wang Y., Margolis A.E., Calafat A.M., Wang S., Garcia W., Hoepner L.A., Peterson B.S., Rauh V. (2016). Bisphenol A exposure and symptoms of anxiety and depression among inner city children at 10–12 years of age. Environ. Res..

[14] Vermeir G., Covaci A., Van Larebeke N., Schoeters G., Nelen V., Koppen G., Viaene M. (2021). Neurobehavioural and cognitive effects of prenatal exposure to organochlorine compounds in three year old children. BMC Pediatr..

[15] Jasarevic E., Williams S.A., Vandas G.M., Ellersieck M.R., Liao C., Kannan K., Roberts R.M., Geary D.C., Rosenfeld C.S. (2013). Sex and dose-dependent effects of developmental exposure to bisphenol A on anxiety and spatial learning in deer mice (*Peromyscus maniculatus* bairdii) offspring. Horm. Behav..

[16] Matsuda S., Matsuzawa D., Ishii D., Tomizawa H., Sutoh C., Nakazawa K., Amano K., Sajiki J., Shimizu E. (2012). Effects of perinatal exposure to low dose of bisphenol A on anxiety like behavior and dopamine metabolites in brain. Prog. Neuro-Psychopharmacol. Biol. Psychiatry.

[17] Patisaul H.B., Bateman H.L. (2008). Neonatal exposure to endocrine active compounds or an ERbeta agonist increases adult anxiety and aggression in gonadally intact male rats. Horm. Behav..

[18] Xu X., Hong X., Xie L., Li T., Yang Y., Zhang Q., Zhang G., Liu X. (2012). Gestational and lactational exposure to bisphenol-A affects anxiety- and depression-like behaviors in mice. Horm. Behav..

[19] Quinnies K.M., Harris E.P., Snyder R.W., Sumner S.S., Rissman E.F. (2017). Direct and transgenerational effects of low doses of perinatal di-(2-ethylhexyl) phthalate (DEHP) on social behaviors in mice. PLoS ONE.

[20] Carbone S., Ponzo O.J., Gobetto N., Samaniego Y.A., Reynoso R., Scacchi P., Moguilevsky J.A., Cutrera R. (2013). Antiandrogenic effect of perinatal exposure to the endocrine disruptor di-(2-ethylhexyl) phthalate increases anxiety-like behavior in male rats during sexual maturation. Horm. Behav..

[21] Bell M.R., Thompson L.M., Rodriguez K., Gore A.C. (2016). Two-hit exposure to polychlorinated biphenyls at gestational and juvenile life stages: 1. Sexually dimorphic effects on social and anxiety-like behaviors. Horm. Behav..

[22] Gillette R., Reilly M.P., Topper V.Y., Thompson L.M., Crews D., Gore A.C. (2017). Anxiety-like behaviors in adulthood are altered in male but not female rats exposed to low dosages of polychlorinated biphenyls in utero. Horm. Behav..

[23] Sobolewski M., Conrad K., Allen J.L., Weston H., Martin K., Lawrence B.P., Cory-Slechta D.A. (2014). Sex-specific enhanced behavioral toxicity induced by maternal exposure to a mixture of low dose endocrine-disrupting chemicals. Neurotoxicology.

[24] Silva E., Rajapakse N., Kortenkamp A. (2002). Something from “nothing”—eight weak estrogenic chemicals combined at concentrations below NOECs produce significant mixture effects. Environ. Sci. Technol..

[25] Conley J.M., Lambright C.S., Evans N., Cardon M., Medlock-Kakaley E., Wilson V.S., Gray L.E. (2021). A mixture of 15 phthalates and pesticides below individual chemical no observed adverse effect levels (NOAELs) produces reproductive tract malformations in the male rat. Environ. Int..

[26] Gaudriault P., Mazaud-Guittot S., Lavoué V., Coiffec I., Lesné L., Dejucq-Rainsford N., Scholze M., Kortenkamp A., Jégou B. (2017). Endocrine Disruption in Human Fetal Testis Explants by Individual and Combined Exposures to Selected Pharmaceuticals, Pesticides, and Environmental Pollutants. Environ. Health Perspect..

[27] Fini J.B., Mughal B.B., Le Mevel S., Leemans M., Lettmann M., Spirhanzlova P., Affaticati P., Jenett A., Demeneix B.A. (2017). Human amniotic fluid contaminants alter thyroid hormone signalling and early brain development in Xenopus embryos. Sci. Rep..

[28] Overgaard A., Holst K., Mandrup K.R., Boberg J., Christiansen S., Jacobsen P.R., Hass U., Mikkelsen J.D. (2013). The effect of perinatal exposure to ethinyl oestradiol or a mixture of endocrine disrupting pesticides on kisspeptin neurons in the rat hypothalamus. Neurotoxicology.

[29] Crofton K.M., Craft E.S., Hedge J.M., Gennings C., Simmons J.E., Carchman R.A., Carter W.H., DeVito M.J. (2005). Thyroid-hormone-disrupting chemicals: Evidence for dose-dependent additivity or synergism. Environ. Health Perspect..

[30] Martin O.V., Evans R.M., Faust M., Kortenkamp A. (2017). A Human Mixture Risk Assessment for Neurodevelopmental Toxicity Associated with Polybrominated Diphenyl Ethers Used as Flame Retardants. Environ. Health Perspect..

[31] Bornehag C.G., Kitraki E., Stamatakis A., Panagiotidou E., Rudén C., Shu H., Lindh C., Ruegg J., Gennings C. (2019). A Novel Approach to Chemical Mixture Risk Assessment-Linking Data from Population-Based Epidemiology and Experimental Animal Tests. Risk Anal..

[32] Schildroth S., Wise L.A., Wesselink A.K., De La Cruz P., Bethea T.N., Weuve J., Fruh V., Botelho J.C., Sjodin A., Calafat A.M. (2021). Correlates of Persistent Endocrine-Disrupting Chemical Mixtures among Reproductive-Aged Black Women. Environ. Sci. Technol..

[33] Champagne F.A., Meaney M.J. (2006). Stress During Gestation Alters Postpartum Maternal Care and the Development of the Offspring in a Rodent Model. Biol. Psychiatry.

[34] Romeo R.D. (2015). Perspectives on stress resilience and adolescent neurobehavioral function. Neurobiol. Stress.

[35] Bell M.R., Hart B.G., Gore A.C. (2016). Two-hit exposure to polychlorinated biphenyls at gestational and juvenile life stages: 2. Sex-specific neuromolecular effects in the brain. Mol. Cell Endocrinol..

[36] Hill R.A., Kiss Von Soly S., Ratnayake U., Klug M., Binder M.D., Hannan A.J., van den Buuse M. (2014). Long-term effects of combined neonatal and adolescent stress on brain-derived neurotrophic factor and dopamine receptor expression in the rat forebrain. Biochim. Biophys. Acta.

[37] Hill R.A., Klug M., Kiss Von Soly S., Binder M.D., Hannan A.J., van den Buuse M. (2014). Sex-specific disruptions in spatial memory and anhedonia in a “two hit“ rat model correspond with alterations in hippocampal brain-derived neurotrophic factor expression and signaling. Hippocampus.

[38] Panagiotidou E., Zerva S., Mitsiou D.J., Alexis M.N., Kitraki E. (2014). Perinatal exposure to low-dose bisphenol A affects the neuroendocrine stress response in rats. J. Endocrinol..

[39] Gillette R., Dias M., Reilly M.P., Thompson L.M., Castillo N.J., Vasquez E.L., Crews D., Gore A.C. (2022). Two Hits of EDCs Three Generations Apart: Effects on Social Behaviors in Rats, and Analysis by Machine Learning. Toxics.

[40] Krishnan K., Mittal N., Thompson Lindsay M., Rodriguez-Santiago M., Duvauchelle Christine L., Crews D., Gore Andrea C. (2018). Effects of the endocrine-disrupting chemicals, vinclozolin and polychlorinated biphenyls, on physiological and sociosexual phenotypes in F2 generation Sprague-Dawley rats. Environ. Health Perspect..

[41] Dickerson S.M., Cunningham S.L., Patisaul H.B., Woller M.J., Gore A.C. (2011). Endocrine disruption of brain sexual differentiation by developmental PCB exposure. Endocrinology.

[42] Arnold A.P., Gorski R.A. (1984). Gonadal steroid induction of structural sex differences in the central nervous system. Ann. Rev. Neurosci..

[43] Szilagyi J.T., Freedman A.N., Kepper S.L., Keshava A.M., Bangma J.T., Fry R.C. (2020). Per- and Polyfluoroalkyl Substances Differentially Inhibit Placental Trophoblast Migration and Invasion In Vitro. Toxicol. Sci..

[44] Björvang R.D., Vinnars M.T., Papadogiannakis N., Gidlöf S., Mamsen L.S., Mucs D., Kiviranta H., Rantakokko P., Ruokojärvi P., Lindh C.H. (2021). Mixtures of persistent organic pollutants are found in vital organs of late gestation human fetuses. Chemosphere.

[45] Mamsen L.S., Björvang R.D., Mucs D., Vinnars M.T., Papadogiannakis N., Lindh C.H., Andersen C.Y., Damdimopoulou P. (2019). Concentrations of perfluoroalkyl substances (PFASs) in human embryonic and fetal organs from first, second, and third trimester pregnancies. Environ. Int..

[46] Gerona R.R., Woodruff T.J., Dickenson C.A., Pan J., Schwartz J.M., Sen S., Friesen M.W., Fujimoto V.Y., Hunt P.A. (2013). Bisphenol-A (BPA), BPA glucuronide, and BPA sulfate in midgestation umbilical cord serum in a northern and central California population. Environ. Sci. Technol..

[47] Sandau C.D., Ayotte P., Dewailly E., Duffe J., Norstrom R.J. (2002). Pentachlorophenol and hydroxylated polychlorinated biphenyl metabolites in umbilical cord plasma of neonates from coastal populations in Quebec. Environ. Health Perspect..

[48] Golub M.S., Donald J.M., Reyes J.A. (1991). Reproductive toxicity of commercial PCB mixtures: LOAELs and NOAELs from animal studies. Environ. Health Perspect..

[49] Tyl R.W., Myers C.B., Marr M.C., Thomas B.F., Keimowitz A.R., Brine D.R., Veselica M.M., Fail P.A., Chang T.Y., Seely J.C. (2002). Three-generation reproductive toxicity study of dietary bisphenol A in CD Sprague-Dawley rats. Toxicol. Sci..

[50] FitzGerald R., Van Loveren H., Civitella C., Castoldi A.F., Bernasconi G., European Food Safety Authority (2020). Assessment of new information on Bisphenol S (BPS) submitted in response to the Decision 1 under REACH Regulation (EC) No 1907/2006. EFSA Supporting Publ..

[51] Blystone C.R., Kissling G.E., Bishop J.B., Chapin R.E., Wolfe G.W., Foster P.M. (2010). Determination of the di-(2-ethylhexyl) phthalate NOAEL for reproductive development in the rat: Importance of the retention of extra animals to adulthood. Toxicol. Sci..

[52] Zhang Y., Jiang X., Chen B. (2004). Reproductive and developmental toxicity in F1 Sprague-Dawley male rats exposed to di-n-butyl phthalate in utero and during lactation and determination of its NOAEL. Reprod. Toxicol..

[53] Luebker D.J., Case M.T., York R.G., Moore J.A., Hansen K.J., Butenhoff J.L. (2005). Two-generation reproduction and cross-foster studies of perfluorooctanesulfonate (PFOS) in rats. Toxicology.

[54] Li P., Ma R., Dong L., Liu L., Zhou G., Tian Z., Zhao Q., Xia T., Zhang S., Wang A. (2019). Autophagy impairment contributes to PBDE-47-induced developmental neurotoxicity and its relationship with apoptosis. Theranostics.

[55] Schantz S.L., Moshtaghian J., Ness D.K. (1995). Spatial learning deficits in adult rats exposed to ortho-substituted PCB congeners during gestation and lactation. Fundam. Appl. Toxicol..

[56] Hellwig J., van Ravenzwaay B., Mayer M., Gembardt C. (2000). Pre- and postnatal oral toxicity of vinclozolin in Wistar and Long-Evans rats. Regul. Toxicol. Pharmacol..

[57] Gillette R., Miller-Crews I., Nilsson E.E., Skinner M.K., Gore A.C., Crews D. (2014). Sexually dimorphic effects of ancestral exposure to vinclozolin on stress reactivity in rats. Endocrinology.

[58] Reilly M.P., Kunkel M.N., Thompson L.M., Zentay A., Weeks C.D., Crews D., Cormack L.K., Gore A.C. (2022). Effects of endocrine-disrupting chemicals on hypothalamic oxytocin and vasopressin systems. J. Exp. Zool. A Ecol. Integr. Physiol..

[59] Pfaffl M.W. (2001). A new mathematical model for relative quantification in real-time RT-PCR. Nucleic Acids Res..

[60] Schmittgen T.D., Livak K.J. (2008). Analyzing real-time PCR data by the comparative C(T) method. Nat. Protoc..

[61] La Merrill M.A., Vandenberg L.N., Smith M.T., Goodson W., Browne P., Patisaul H.B., Guyton K.Z., Kortenkamp A., Cogliano V.J., Woodruff T.J. (2020). Consensus on the key characteristics of endocrine-disrupting chemicals as a basis for hazard identification. Nat. Rev. Endocrinol..

[62] Zhou C., Gao L., Flaws J.A. (2017). Prenatal exposure to an environmentally relevant phthalate mixture disrupts reproduction in F1 female mice. Toxicol. Appl. Pharmacol..

[63] Kundakovic M., Gudsnuk K., Franks B., Madrid J., Miller R.L., Perera F.P., Champagne F.A. (2013). Sex-specific epigenetic disruption and behavioral changes following low-dose in utero bisphenol A exposure. Proc. Natl. Acad. Sci. USA.

[64] Grønnestad R., Johanson S.M., Müller M.H.B., Schlenk D., Tanabe P., Krøkje Å., Jaspers V.L.B., Jenssen B.M., Ræder E.M., Lyche J.L. (2021). Effects of an environmentally relevant PFAS mixture on dopamine and steroid hormone levels in exposed mice. Toxicol. Appl. Pharmacol..

[65] Hernandez Scudder M.E., Young R.L., Thompson L.M., Kore P., Crews D., Hofmann H.A., Gore A.C. (2021). EDCs Reorganize Brain-Behavior Phenotypic Relationships in Rats. J. Endocr. Soc..

[66] Hernandez Scudder M.E., Weinberg A., Thompson L., Crews D., Gore A.C. (2020). Prenatal EDCs impair mate and odor preference and activation of the VMN in male and female rats. Endocrinology.

[67] Wang A., Padula A., Sirota M., Woodruff T.J. (2016). Environmental influences on reproductive health: The importance of chemical exposures. Fertil. Steril..

[68] Ikezuki Y., Tsutsumi O., Takai Y., Kamei Y., Taketani Y. (2002). Determination of bisphenol A concentrations in human biological fluids reveals significant early prenatal exposure. Hum. Reprod..

[69] Watkins D.J., Téllez-Rojo M.M., Ferguson K.K., Lee J.M., Solano-Gonzalez M., Blank-Goldenberg C., Peterson K.E., Meeker J.D. (2014). In utero and peripubertal exposure to phthalates and BPA in relation to female sexual maturation. Environ. Res..

[70] Haines D.A., Murray J. (2012). Human biomonitoring of environmental chemicals--early results of the 2007–2009 Canadian Health Measures Survey for males and females. Int. J. Hyg. Environ. Health.

[71] Li N., Papandonatos G.D., Calafat A.M., Yolton K., Lanphear B.P., Chen A., Braun J.M. (2020). Gestational and childhood exposure to phthalates and child behavior. Environ. Int..

[72] Wickerham E.L., Lozoff B., Shao J., Kaciroti N., Xia Y., Meeker J.D. (2012). Reduced birth weight in relation to pesticide mixtures detected in cord blood of full-term infants. Environ. Int..

[73] Doremus-Fitzwater T.L., Varlinskaya E.I., Spear L.P. (2009). Social and non-social anxiety in adolescent and adult rats after repeated restraint. Physiol. Behav..

[74] Sandi C., Haller J. (2015). Stress and the social brain: Behavioural effects and neurobiological mechanisms. Nat. Rev. Neurosci..

[75] Layton A.C., Sanseverino J., Gregory B.W., Easter J.P., Sayler G.S., Schultz T.W. (2002). In vitro estrogen receptor binding of PCBs: Measured activity and detection of hydroxylated metabolites in a recombinant yeast assay. Toxicol. Appl. Pharmacol..

[76] Wrobel M., Kaminski K., Kotwica J. (2005). In vitro effects of polychlorinated biphenyls (PCBs) on the contractility of bovine myometrium from the periovulatory stage of the estrous cycle. Reprod. Biol..

[77] Kavlock R., Cummings A. (2005). Mode of action: Inhibition of androgen receptor function--vinclozolin-induced malformations in reproductive development. Crit. Rev. Toxicol..

[78] Mylchreest E., Sar M., Cattley R.C., Foster P.M. (1999). Disruption of androgen-regulated male reproductive development by di(n-butyl) phthalate during late gestation in rats is different from flutamide. Toxicol. Appl. Pharmacol..

[79] Kirk A.B., Michelsen-Correa S., Rosen C., Martin C.F., Blumberg B. (2021). PFAS and Potential Adverse Effects on Bone and Adipose Tissue Through Interactions With PPARγ. Endocrinology.

[80] Petrulis J.R., Bunce N.J. (2000). Competitive behavior in the interactive toxicology of halogenated aromatic compounds. J. Biochem. Mol. Toxicol..

[81] Song J., Li Y., Zhao C., Zhou Q., Zhang J. (2021). Interaction of BDE-47 with nuclear receptors (NRs) based on the cytotoxicity: In vitro investigation and molecular interaction. Ecotoxicol. Environ. Saf..

[82] Behr A.C., Plinsch C., Braeuning A., Buhrke T. (2020). Activation of human nuclear receptors by perfluoroalkylated substances (PFAS). Toxicol. Vitr..

[83] Cao H., Wang L., Liang Y., Li Z., Feng H., Sun Y., Zhang A., Fu J. (2019). Protonation state effects of estrogen receptor α on the recognition mechanisms by perfluorooctanoic acid and perfluorooctane sulfonate: A computational study. Ecotoxicol. Environ. Saf..

[84] Qu J., Han Y., Zhao Z., Wu Y., Lu Y., Chen G., Jiang J., Qiu L., Gu A., Wang X. (2021). Perfluorooctane sulfonate interferes with non-genomic estrogen receptor signaling pathway, inhibits ERK1/2 activation and induces apoptosis in mouse spermatocyte-derived cells. Toxicology.

[85] Qin W.P., Cao L.Y., Li C.H., Guo L.H., Colbourne J., Ren X.M. (2020). Perfluoroalkyl Substances Stimulate Insulin Secretion by Islet β Cells via G Protein-Coupled Receptor 40. Environ. Sci. Technol..

[86] Mohajer N., Du C.Y., Checkcinco C., Blumberg B. (2021). Obesogens: How They Are Identified and Molecular Mechanisms Underlying Their Action. Front. Endocrinol..

[87] Sharma P., Mandal M.B., Katiyar R., Singh S.P., Birla H. (2021). A Comparative Study of Effects of 28-Day Exposure of Bisphenol A and Bisphenol S on Body Weight Changes, Organ Histology, and Relative Organ Weight. Int. J. Appl. Basic. Med. Res..

[88] Tatsuta N., Kurokawa N., Nakai K., Suzuki K., Iwai-Shimada M., Murata K., Satoh H. (2017). Effects of intrauterine exposures to polychlorinated biphenyls, methylmercury, and lead on birth weight in Japanese male and female newborns. Environ. Health Prev. Med..

[89] Robledo C.A., Yeung E., Mendola P., Sundaram R., Maisog J., Sweeney A.M., Barr D.B., Louis G.M. (2015). Preconception maternal and paternal exposure to persistent organic pollutants and birth size: The LIFE study. Environ. Health Perspect..

[90] Gray L.E., Ostby J.S., Kelce W.R. (1994). Developmental effects of an environmental antiandrogen: The fungicide vinclozolin alters sex differentiation of the male rat. Toxicol. Appl. Pharmacol..

[91] Lopez-Rodriguez D., Franssen D., Heger S., Parent A.S. (2021). Endocrine-disrupting chemicals and their effects on puberty. Best Pract. Res. Clin. Endocrinol. Metab..

[92] Dickerson S.M., Gore A.C. (2007). Estrogenic environmental endocrine-disrupting chemical effects on reproductive neuroendocrine function and dysfunction across the life cycle. Rev. Endocr. Metab. Disord..

[93] Sun Y., Fang J., Wan Y., Su P., Tao F. (2019). Role of polygenic risk in susceptibility to accelerated pubertal onset following chronic stress exposure. Eur. J. Endocrinol..

[94] Gur R.E., Moore T.M., Rosen A.F.G., Barzilay R., Roalf D.R., Calkins M.E., Ruparel K., Scott J.C., Almasy L., Satterthwaite T.D. (2019). Burden of Environmental Adversity Associated With Psychopathology, Maturation, and Brain Behavior Parameters in Youths. JAMA Psychiatry.

[95] Cowan C.S.M., Richardson R. (2019). Early-life stress leads to sex-dependent changes in pubertal timing in rats that are reversed by a probiotic formulation. Dev. Psychobiol..

[96] Manzano Nieves G., Schilit Nitenson A., Lee H.I., Gallo M., Aguilar Z., Johnsen A., Bravo M., Bath K.G. (2019). Early Life Stress Delays Sexual Maturation in Female Mice. Front. Mol. Neurosci..

[97] Zhang L., Zhang D., Sun Y. (2019). Adverse Childhood Experiences and Early Pubertal Timing Among Girls: A Meta-Analysis. Int. J. Environ. Res. Public Health.

[98] Walley S.N., Krumm E.A., Yasrebi A., Wiersielis K.R., O’Leary S., Tillery T., Roepke T.A. (2021). Maternal organophosphate flame-retardant exposure alters offspring feeding, locomotor and exploratory behaviors in a sexually-dimorphic manner in mice. J. Appl. Toxicol..

[99] Kaur S., Kinkade J.A., Green M.T., Martin R.E., Willemse T.E., Bivens N.J., Schenk A.K., Helferich W.G., Trainor B.C., Fass J. (2021). Disruption of global hypothalamic microRNA (miR) profiles and associated behavioral changes in California mice (Peromyscus californicus) developmentally exposed to endocrine disrupting chemicals. Horm. Behav..

[100] Wolstenholme J.T., Edwards M., Shetty S.R., Gatewood J.D., Taylor J.A., Rissman E.F., Connelly J.J. (2012). Gestational exposure to bisphenol A produces transgenerational changes in behaviors and gene expression. Endocrinology.

[101] Hodes G.E., Pfau M.L., Purushothaman I., Ahn H.F., Golden S.A., Christoffel D.J., Magida J., Brancato A., Takahashi A., Flanigan M.E. (2015). Sex Differences in Nucleus Accumbens Transcriptome Profiles Associated with Susceptibility versus Resilience to Subchronic Variable Stress. J. Neurosci..

[102] Bale T.L., Epperson C.N. (2015). Sex differences and stress across the lifespan. Nat. Neurosci..

[103] Wang D.M., Zhang J.J., Huang Y.B., Zhao Y.Z., Sui N. (2019). Peripubertal stress of male, but not female rats increases morphine-induced conditioned place preference and locomotion in adulthood. Dev. Psychobiol..

[104] Smith B.L., Morano R.L., Ulrich-Lai Y.M., Myers B., Solomon M.B., Herman J.P. (2018). Adolescent environmental enrichment prevents behavioral and physiological sequelae of adolescent chronic stress in female (but not male) rats. Stress.

[105] Kaplowitz E.T., Savenkova M., Karatsoreos I.N., Romeo R.D. (2016). Somatic and Neuroendocrine Changes in Response to Chronic Corticosterone Exposure During Adolescence in Male and Female Rats. J. Neuroendocrinol..

[106] Adam N., Brusamonti L., Mhaouty-Kodja S. (2021). Exposure of Adult Female Mice to Low Doses of di(2-ethylhexyl) Phthalate Alone or in an Environmental Phthalate Mixture: Evaluation of Reproductive Behavior and Underlying Neural Mechanisms. Environ. Health Perspect..

[107] Dombret C., Capela D., Poissenot K., Parmentier C., Bergsten E., Pionneau C., Chardonnet S., Hardin-Pouzet H., Grange-Messent V., Keller M. (2017). Neural Mechanisms Underlying the Disruption of Male Courtship Behavior by Adult Exposure to Di(2-ethylhexyl) Phthalate in Mice. Environ. Health Perspect..

[108] Gore A.C., Holley A.M., Crews D. (2018). Mate choice, sexual selection, and endocrine-disrupting chemicals. Horm. Behav..

[109] Patisaul H.B., Sullivan A.W., Radford M.E., Walker D.M., Adewale H.B., Winnik B., Coughlin J.L., Buckley B., Gore A.C. (2012). Anxiogenic effects of developmental bisphenol A exposure are associated with gene expression changes in the juvenile rat amygdala and mitigated by soy. PLoS ONE.

[110] Cao J., Joyner L., Mickens J.A., Leyrer S.M., Patisaul H.B. (2014). Sex-specific Esr2 mRNA expression in the rat hypothalamus and amygdala is altered by neonatal bisphenol A exposure. Reproduction.

[111] Gillette R., Son M.J., Ton L., Gore A.C., Crews D. (2018). Passing experiences on to future generations: Endocrine disruptors and transgenerational inheritance of epimutations in brain and sperm. Epigenetics.

[112] Hatcher K.M., Willing J., Chiang C., Rattan S., Flaws J.A., Mahoney M.M. (2019). Exposure to di-(2-ethylhexyl) phthalate transgenerationally alters anxiety-like behavior and amygdala gene expression in adult male and female mice. Physiol. Behav..

[113] Viau V. (2002). Functional cross-talk between the hypothalamic-pituitary-gonadal and -adrenal axes. J. Neuroendocrinol..

[114] Rivest S., Rivier C. (1991). Influence of the paraventricular nucleus of the hypothalamus in the alteration of neuroendocrine functions induced by intermittent footshock or interleukin. Endocrinology.

