# Supplementary material for: Prenatal Exposure to an EDC Mixture, NeuroMix: Effects on Brain, Behavior, and Stress Responsiveness in Rats
Source: Toxics. 2022 Mar 3;10(3):122. doi: 10.3390/toxics10030122 (PMC8954446; doi:10.3390/toxics10030122)
Supplement: Supplementary file 1 [file toxics-10-00122-s001.zip › toxics-1562212-supplementary.pdf]

toxics MDPI

Supplementary Materials: Prenatal Exposure to an EDC Mixture, NeuroMix: Effects on Brain, Behavior, and Stress Responsiveness in Rats

Andrea C. Gore, Tatum Moore, Matthew J. Groom and Lindsay M. Thompson

Table S1. Statistical analyses.

Development and Physiology		Females			
		Test	*p*-value	η_p^2 for ANOVA ϵ^2 for KW	F(df) for ANOVA x^2 for KW
P1 Body Weight	Treatment	ANOVA	0.070	0.071	F (1, 45) =1.859
P14 Body Weight	Treatment	ANOVA	0.828	13.120	F (1, 94) = 0.047
	Stress		0.124	664.900	F (1, 94) = 2.414
	TxS		0.344	249.200	F (1, 94) = 0.905
P1 AGI	Treatment	ANOVA	0.030	0.101	F (1, 45) =2.245
P14 AGI	Treatment	ANOVA	0.214	0.034	F (1, 45) =1.262

Development and Physiology		Males			
		Test	*p*-value	η_p^2 for ANOVA ϵ^2 for KW	F(df) for ANOVA x^2 for KW
P1 Body Weight	Treatment	ANOVA	0.001	0.189	F (1, 48) = 3.347
P84 Body Weight	Treatment	ANOVA	0.077	2709.000	F (1, 97) = 3.190
	Stress		0.133	1950.000	F (1, 97) = 2.296
	TxS		0.508	374.400	F (1, 97) = 0.441
P1 AGI	Treatment	ANOVA	0.207	0.033	F (1, 48) =1.280
P14 AGI	Treatment	ANOVA	0.000	0.371	F (1, 48) =5.323

Light Dark Box		Females			
		Test	*p*-value	η_p^2 for ANOVA ϵ^2 for KW	F(df) for ANOVA x^2 for KW
Distance	Treatment	ANOVA	0.373	0.008	F(1,94)=0.801
	Stress		0.853	0.000	F(1,94)=0.035
	TxS		0.754	0.001	F(1,94)=0.099
Freezing Episodes	Treatment	KW	0.806	0.001	$x^2(1)=0.06$
	Stress		0.095	0.029	$x^2(1)=2.787$
Time Freezing	Treatment	KW	0.601	0.003	$x^2(1)=0.274$
	Stress		0.066	0.035	$x^2(1)=3.386$
Light Time	Treatment	KW	0.029	0.049	$x^2(1)=4.776$
	Stress		0.708	0.001	$x^2(1)=0.14$
Light Freezing Episodes	Treatment	KW	0.037	0.045	$x^2(1)=4.331$
	Stress		0.316	0.010	$x^2(1)=1.004$
Light Freezing Time	Treatment	KW	0.177	0.019	$x^2(1)=1.82$
	Stress		0.399	0.007	$x^2(1)=0.71$
Dark Time	Treatment	KW	0.028	0.050	$x^2(1)=4.807$
	Stress		0.708	0.001	$x^2(1)=0.14$
Dark Freezing Episodes	Treatment	ANOVA	0.379	0.008	F(1,94)=0.780
	Stress		0.123	0.025	F(1,94)=2.420
	TxS		0.361	0.009	F(1,94)=0.844
Dark Freezing Time	Treatment	KW	0.284	0.012	$x^2(1)=1.148$
	Stress		0.211	0.016	$x^2(1)=1.561$

Speed	Treatment	ANOVA	0.369	0.009	F(1,94)=0.814
	Stress		0.843	0.000	F(1,94)=0.039
	TxS		0.740	0.001	F(1,94)=0.111
Line Crossing	Treatment	KW	0.643	0.002	x^2(1)=0.215
	Stress		0.977	0.000	x^2(1)=0.001

**Light Dark Box**			**Males**		
		Test	*p*-value	η_P^2 for ANOVA ϵ^2 for KW	F(df) for ANOVA x^2 for KW
Distance	Treatment	ANOVA	0.210	0.016	F(1,97)=1.596
	Stress		0.752	0.001	F(1,97)=0.101
	TxS		0.736	0.001	F(1,97)=0.114
Freezing Episodes	Treatment	KW	0.187	0.017	x^2(1)=1.741
	Stress		0.067	0.033	x^2(1)=3.344
Time Freezing	Treatment	KW	0.372	0.008	x^2(1)=0.798
	Stress		0.012	0.063	x^2(1)=6.267
Light Time	Treatment	KW	0.421	0.006	x^2(1)=0.648
	Stress		0.347	0.009	x^2(1)=0.885
Light Freezing Episodes	Treatment	KW	0.723	0.001	x^2(1)=0.126
	Stress		0.986	0.000	x2(1)=0.000
Light Freezing Time	Treatment	KW	0.892	0.000	x^2(1)=0.019
	Stress		0.937	0.000	x^2(1)=0.006
Dark Time	Treatment	KW	0.455	0.006	x^2(1)=0.559
	Stress		0.313	0.010	x^2(1)=1.019
Dark Freezing Episodes	Treatment	KW	0.229	0.014	x^2(1)=1.449
	Stress		0.189	0.017	x^2(1)=1.724
Dark Freezing Time	Treatment	KW	0.557	0.003	x^2(1)=0.346
	Stress		0.035	0.044	x^2(1)=4.433
Speed	Treatment	ANOVA	0.224	0.015	F(1,97)=1.500
	Stress		0.748	0.001	F(1,97)=0.104
	TxS		0.725	0.001	F(1,97)=0.125
Line Crossing	Treatment	KW	0.349	0.009	x^2(1)=0.876
	Stress		0.051	0.038	x^2(1)=3.803

**Sociability**			**Females**		
		Test	*p*-value	η_P^2 for ANOVA ϵ^2 for KW	F(df) for ANOVA x^2 for KW
Distance Traveled	Treatment	ANOVA	0.038	0.046	F(1,92)=4.441
	Stress		0.541	0.004	F(1,92)=0.376
	TxS		0.769	0.001	F(1,92)=0.087
Time in Center	Treatment	KW	0.015	0.062	x^2(1)=5.875
	Stress		0.362	0.009	x^2(1)=0.830
Time Near Social	Treatment	KW	0.059	0.037	x^2(1)=3.553
	Stress		0.725	0.001	x^2(1)=0.124
Avg Visit Time to Social	Treatment	KW	0.029	0.050	x^2(1)=4.794
	Stress		0.956	0.000	x^2(1)=0.003
Time Near Empty	Treatment	KW	0.214	0.016	x^2(1)=1.545
	Stress		0.468	0.006	x^2(1)=0.527
Avg Visit Time to Empty	Treatment	KW	0.912	0.000	x^2(1)=0.012
	Stress		0.662	0.002	x^2(1)=0.191
Social Pref Score	Treatment	KW	0.061	0.037	x^2(1)=3.498
	Stress		0.706	0.002	x^2(1)=0.143

**Sociability**	**Males**

		Test	*p*-value	η_p^2 for ANOVA ϵ^2 for KW	F(df) for ANOVA x^2 for KW
Distance Traveled	Treatment	KW	0.373	0.008	$x^2(1)=0.792$
	Stress		0.096	0.028	$x^2(1)=2.763$
Time in Center	Treatment	KW	0.216	0.015	$x^2(1)=1.534$
	Stress		0.463	0.005	$x^2(1)=0.538$
Time Near Social	Treatment	ANOVA	0.781	0.001	$F(1,96)=0.078$
	Stress		0.564	0.004	$F(1,96)=0.336$
	Treatment:Stress		0.516	0.004	$F(1,96)=0.426$
Avg Visit Time to Social	Treatment	KW	0.532	0.004	$x^2(1)=0.390$
	Stress		0.021	0.053	$x^2(1)=5.296$
Time Near Empty	Treatment	KW	0.796	0.677	$x^2(1)=67.0$
	Stress		0.050	0.039	$x^2(1)=3.838$
Avg Visit Time to Empty	Treatment	KW	0.405	0.007	$x^2(1)=0.694$
	Stress		0.006	0.075	$x^2(1)=7.471$
Social Pref Score	Treatment	KW	0.709	0.001	$x^2(1)=0.139$
	Stress		0.670	0.002	$x^2(1)=0.181$

**Social Novelty**			**Females**		
		Test	*p*-value	η_p^2 for ANOVA ϵ^2 for KW	F(df) for ANOVA x^2 for KW
Distance Traveled	Treatment	ANOVA	0.247	0.015	$F(1,92)=1.360$
	Stress		0.629	0.003	$F(1,92)=0.236$
	Treatment:Stress		0.906	0.000	$F(1,92)=0.014$
Time in Center	Treatment	ANOVA	0.219	0.016	$F(1,92)=1.532$
	Stress		0.113	0.027	$F(1,92)=2.561$
	Treatment:Stress		0.556	0.004	$F(1,92)=0.350$
Time Near Familiar	Treatment	KW	0.883	0.009	$x^2(1)=0.022$
	Stress		0.097	0.001	$x^2(1)=2.753$
Avg Visit Time to Familiar	Treatment	KW	0.161	0.002	$x^2(1)=1.963$
	Stress		0.252	0.003	$x^2(1)=1.310$
Time Near Novel	Treatment	KW	0.974	0.010	$x^2(1)=0.001$
	Stress		0.196	0.002	$x^2(1)=1.671$
Avg Visit Time to Novel	Treatment	KW	0.953	0.010	$x^2(1)=0.003$
	Stress		0.019	0.000	$x^2(1)=2.865$
Social Novelty Score	Treatment	ANOVA	0.331	0.010	$F(1,92)=0.956$
	Stress		0.026	0.053	$F(1,92)=5.105$
	Treatment:Stress		0.098	0.029	$F(1,92)=2.791$

**Social Novelty**			**Males**		
		Test	*p*-value	η_p^2 for ANOVA ϵ^2 for KW	F(df) for ANOVA x^2 for KW
Distance Traveled	Treatment	ANOVA	0.796	0.001	$F(1,96)=0.067$
	Stress		0.582	0.003	$F(1,96)=0.305$
	Treatment:Stress		0.317	0.010	$F(1,96)=1.012$
Time in Center	Treatment	KW	0.612	0.003	$x^2(1)=0.257$
	Stress		0.917	0.000	$x^2(1)=0.011$
Time Near Familiar	Treatment	KW	0.379	0.008	$x^2(1)=0.774$
	Stress		0.198	0.017	$x^2(1)=1.660$
Avg Visit Time to Familiar	Treatment	KW	0.939	0.000	$x^2(1)=0.006$
	Stress		0.557	0.003	$x^2(1)=0.344$
Time Near Novel	Treatment	KW	0.769	0.001	$x^2(1)=0.086$

	Stress		0.161	0.020	$x^2(1)=1.961$
Avg Visit Time to Novel	Treatment	KW	0.301	0.011	$x^2(1)=1.071$
	Stress		0.579	0.003	$x^2(1)=0.308$
Social Novelty Score	Treatment	ANOVA	0.470	0.005	F(1,96)=0.527
	Stress		0.104	0.027	F(1,96)=2.696
	Treatment:Stress		0.528	0.004	F(1,96)=0.401

**Mate Preference**			**Females**		
		Test	*p*-value	η_p^2 for ANOVA ϵ^2 for KW	F(df) for ANOVA x^2 for KW
Distance	Treatment	ANOVA	0.003	0.092	F(1,94)=9.542
	Stress		0.170	0.020	F(1,94)=1.911
	Treatment:Stress		0.083	0.032	F(1,94)=3.066
Time Near Hormone	Treatment	ANOVA	0.049	0.041	F(1,94)=3.964
	Stress		0.674	0.002	F(1,94)=0.179
	Treatment:Stress		0.291	0.012	F(1,94)=1.130
Time Near NH	Treatment	ANOVA	0.629	0.003	F(1,94)=0.235
	Stress		0.066	0.036	F(1,94)=3.463
	Treatment:Stress		0.276	0.013	F(1,94)=1.20
Stim Explore Time Hormone	Treatment	KW	0.036	0.045	$x^2(1)=4.410$
	Stress		0.362	0.009	$x^2(1)=0.830$
Nose Touch Number Hormone	Treatment	KW	0.672	0.002	$x^2(1)=0.180$
	Stress		0.057	0.037	$x^2(1)=3.637$
Nose Touch Time Hormone	Treatment	KW	0.722	0.001	$x^2(1)=0.127$
	Stress		0.070	0.034	$x^2(1)=3.294$
Stim Explore Time NH	Treatment	KW	0.409	0.007	$x^2(1)=0.682$
	Stress		0.266	0.013	$x^2(1)=1.238$
Nose Touch Number NH	Treatment	KW	0.926	0.000	$x^2(1)=0.009$
	Stress		0.090	0.030	$x^2(1)=2.879$
Nose Touch Time NH	Treatment	KW	0.822	0.001	$x^2(1)=0.050$
	Stress		0.126	0.024	$x^2(1)=2.336$
Time Near Both Rats	Treatment	ANOVA	0.025	0.053	F(1,94)=5.216
	Stress		0.500	0.005	F(1,94)=0.459
	Treatment:Stress		0.677	0.002	F(1,94)=0.175
Mate Pref Score	Treatment	ANOVA	0.438	0.006	F(1,94)=0.605
	Stress		0.154	0.022	F(1,94)=2.069
	Treatment:Stress		0.170	0.020	F(1,94)=1.908

**Mate Preference**			**Males**		
		Test	*p*-value	η_p^2 for ANOVA ϵ^2 for KW	F(df) for ANOVA x^2 for KW
Distance	Treatment	KW	0.081	0.031	$x^2(1)=3.052$
	Stress		0.763	0.001	$x^2(1)=0.091$
Time Near Hormone	Treatment	ANOVA	0.738	0.001	F(1,95)=0.113
	Stress		0.098	0.029	F(1,95)=2.802
	Treatment:Stress		0.553	0.004	F(1,95)=0.354
Time Near NH	Treatment	KW	0.307	0.011	$x^2(1)=1.054$
	Stress		0.591	0.003	$x^2(1)=0.290$
Stim Explore Time Hormone	Treatment	ANOVA	0.989	0.000	F(1,95)=0.000
	Stress		0.011	0.067	F(1,95)=6.764
	Treatment:Stress		0.606	0.003	F(1,95)=0.269
Nose Touch Number Hormone	Treatment	KW	0.441	0.006	$x^2(1)=0.594$
	Stress		0.039	0.044	$x^2(1)=4.266$
Nose Touch Time Hormone	Treatment	KW	0.355	0.009	$x^2(1)=0.854$

	Stress		0.010	0.069	$x^2(1)$=6.715
Stim Explore Time NH	Treatment	KW	0.092	0.029	$x^2(1)$=2.835
	Stress		0.492	0.005	$x^2(1)$=0.472
Nose Touch Number NH	Treatment	KW	0.466	0.005	$x^2(1)$=0.532
	Stress		0.058	0.037	$x^2(1)$=3.600
Nose Touch Time NH	Treatment	KW	0.964	0.000	$x^2(1)$=0.002
	Stress		0.032	0.047	$x^2(1)$4.605
Time Near Both Rats	Treatment	ANOVA	0.758	0.001	F(1,94)=0.095
	Stress		0.072	0.034	F(1,94)=3.307
	Treatment:Stress		0.855	0.000	F(1,94)=0.034
Mate Pref Score	Treatment	ANOVA	0.461	0.006	F(1,94)=0.548
	Stress		0.343	0.009	F(1,94)=0.909
	Treatment:Stress		0.300	0.011	F(1,94)=1.084

**Gene Expression**		**Females**			
		Test	*p*-value	η_P^2 for ANOVA ϵ^2 for KW	F(df) for ANOVA x^2 for KW
MeA *Ar*	Treatment	ANOVA	0.760	0.003	F (1, 43) = 0.094
	Stress		0.474	0.016	F (1, 43) = 0.521
	Treatment:Stress		0.793	0.002	F (1, 43) = 0.067
MeA *Esr2*	Treatment	ANOVA	0.624	0.050	F (1, 43) = 0.244
	Stress		0.775	0.017	F (1, 43) = 0.086
	Treatment:Stress		0.854	0.007	F (1, 43) = 0.034
PVN *Ar*	Treatment	ANOVA	0.436	0.031	F (1, 42) = 0.620
	Stress		0.144	0.110	F (1, 42) = 2.212
	Treatment:Stress		0.020	0.290	F (1, 42) = 5.811
PVN *Esr2*	Treatment	ANOVA	0.616	0.034	F (1, 42) = 0.256
	Stress		0.991	0.000	F (1, 42) = 0.000
	Treatment:Stress		0.290	0.155	F (1, 42) = 1.151

**Gene Expression**		**Males**			
		Test	*p*-value	η_P^2 for ANOVA ϵ^2 for KW	F(df) for ANOVA x^2 for KW
MeA *Ar*	Treatment	ANOVA	0.815	0.001	F (1, 43) = 0.055
	Stress		0.114	0.054	F (1, 43) = 2.598
	Treatment:Stress		0.027	0.109	F (1, 43) = 5.267
MeA *Esr2*	Treatment	ANOVA	0.012	1.247	F (1, 44) = 6.848
	Stress		0.657	0.037	F (1, 44) = 0.201
	Treatment:Stress		0.357	0.158	F (1, 44) = 0.867
PVN *Ar*	Treatment	ANOVA	0.054	0.340	F (1, 44) = 3.938
	Stress		0.158	0.179	F (1, 44) = 2.067
	Treatment:Stress		0.167	0.170	F (1, 44) = 1.972
PVN *Esr2*	Treatment	ANOVA	0.319	0.179	F (1, 44) = 1.015
	Stress		0.515	0.076	F (1, 44) = 0.432
	Treatment:Stress		0.340	0.164	F (1, 44) = 0.932

Note: P, postnatal day; NH, no hormone treatment; PVN, paraventricular nucleus; MeA, medial amygdala; AGI, anogenital index; Avg, average; KW, Kruskal-Wallis test;.
